# Supplementary material for: Respiratory Health Inequities among Children and Young Adults with Cerebral Palsy in Aotearoa New Zealand: A Data Linkage Study
Source: J Clin Med. 2022 Nov 25;11(23):6968. doi: 10.3390/jcm11236968 (PMC9739165; doi:10.3390/jcm11236968)
Supplement: Supplementary file 1 [file jcm-11-06968-s001.zip › jcm-1988145-supplementary.pdf]

Supplementary Table S1. Antibiotics chosen to represent treatment for respiratory illness by chemical formulation name.

**Chemical name**
Amoxicillin
Amoxicillin with clavulanic acid
Azithromycin
Cefalexin
Cefaclor monohydrate
Ciprofloxacin
Colistin sulphomethate
Doxycycline
Erythromycin ethyl succinate
Flucloxacillin
Roxithromycin
Trimethoprim
Trimethoprim with sulpha-methoxazole
